# Estrogen stimulates SREBP2 expression in hepatic cell lines via an estrogen response element in the SREBP2 promoter

**DOI:** 10.1186/s11658-019-0194-5

**Published:** 2019-12-03

**Authors:** Ye Meng, Lu Zong

**Affiliations:** The First Affiliated Hospital of USTC, Division of Life Sciences and Medicine, University of Science and Technology of China, Hefei, Anhui 230001 People’s Republic of China

**Keywords:** Sterol regulatory element-binding protein 2, Estradiol, Transcription regulation, Lipid metabolism

## Abstract

**Objective:**

Hypoestrogenism in women is strongly associated with menopause and it can lead to lipid disorder, which predisposes people to premature cardiovascular disease. However, the mechanism of lipid disorder remains unclear. Sterol regulatory element-binding protein 2 (SREBP2) is the key transcription factor regulating cholesterol metabolism. We hypothesize that estrogen regulates SREBP2 transcription through an estrogen response element (ERE) in the SREBP2 promoter region.

**Methods:**

Human hepatoblastoma cells (HepG2) were treated with dose-dependent concentrations of estradiol (E_2_) for 24 h. Then, SREBP2 expression was determined via real-time PCR and immunofluorescence. The expressions of the SREBP2 downstream target genes HMGCR and LDLR were determined via real-time PCR. Lipid secretion in the culture media of HepG2 cells was measured using ELISA. Through bioinformatics analysis, we identified high-scoring ERE-like sequences in the SREBP2 gene promoter. Chromatin immunoprecipitation analysis was used to confirm the ERE. DNA fragments of the putative or mutated ERE-like sequence were synthesized and ligated into pGL3-basic plasmid to construct the SREBP2 promoter luciferase reporter systems. SREBP2-Luciferase (SREBP2-Luc), SREBP2-Mutation (SREBP2-Mut) and the blank control were transfected into hepatic cell lines. Luciferase activities were measured using the dual-luciferase reporter assay system. Chromatin immunoprecipitation analysis and the luciferase reporter assay were repeated in human hepatoma cells (HuH-7).

**Results:**

We found that E_2_ dose-dependently increased the expression of SREBP2 in HepG2 cells and that the increased levels were blocked when treated with an estrogen receptor-alpha antagonist. Additionally, E_2_ increased both HMGCR and LDLR expression and lipid secretion in HepG2 cells. Notably, we identified a functional ERE in the SREBP2 gene promoter, to which E_2_ could specifically bind and induce transcription.

**Conclusions:**

An ERE was identified in the SREBP2 gene promoter. It mediates the regulation of SREBP2 expression by estrogen in hepatocytes. This study provides a mechanism to link cardiovascular disease with estrogen.

## Introduction

Effective methods to prevent cardiovascular diseases are essential, since they are major causes of morbidity and mortality around the world [1]. Examples include coronary artery disease, congestive heart failure, peripheral vascular disease, cerebrovascular disease and left ventricular hypertrophy [2].

A large number of epidemiological studies have shown that determinants of cardiovascular disease include behavior, environmental factors and heredity factors [3]. Risk factors of cardiovascular disease include cholesterol level, body mass index (BMI), blood pressure and fasting plasma glucose [4]. Dyslipidemia is an important underlying risk factor, especially in terms of elevated total cholesterol (TC) and low-density lipoprotein cholesterol (LDL-C) levels [5, 6]. LDL-C is the current primary therapeutic target, and lowering its levels, most commonly by treatment with statins, is the current prevention approach. In addition, decreased total cholesterol and triglycerides (TG) are emerging as reliable therapeutic targets of cardiovascular disease [7, 8].

The incidence of cardiovascular disease increases sharply in females after menopause. Studies have shown that this can be mainly attributed to lipid disorder, vascular stability destruction and blood pressure increase, which result from a series of pathological changes caused by decreased estrogen levels [9–11].

As an important steroid hormone, estrogen mainly regulates estrogen-sensitive genes via the classical pathway: estrogen receptor α (ERα) binds to estrogen response elements (EREs) to regulate gene transcription [12]. Estrogen is involved in the functional regulation of multiple organs and systems, and its role in the progression of cardiovascular disease has attracted much attention in recent years. Studies have indicated that estrogen can regulate lipid homeostasis in the adipose tissue, liver and brain, as well as prevent metabolic dyslipidemia [13]. Furthermore, clinical evidence demonstrates that estrogen replacement therapy can reduce the risk of cardiovascular disease in postmenopausal women by improving lipid metabolism [14, 15]. Further studies are needed to identify the mechanisms by which estrogen regulates lipid metabolism and delays the development of cardiovascular disease in postmenopausal women.

Sterol regulatory element-binding proteins (SREBPs) are a family of key nuclear transcription factors that can regulate lipid metabolism by controlling the expression of a series of enzymes required for the synthesis of endogenous cholesterol, triacylglycerol, fatty acid and phospholipid [16]. Nuclear SREBPs activate lipid metabolism-related enzymes by binding to specific sterol regulatory elements (SREs) in the promoters of target genes [17]. There are three isoforms: SREBP1a, SREBP1c and SREBP2. Each plays a different role in lipid synthesis. SREBP1a is the master regulator of lipogenesis, especially in fatty acid and triglyceride biosynthesis. SREBP1c regulates fatty acid synthesis and insulin-induced glucose homeostasis. SREBP2 is a crucial factor for (and relatively specific to) cholesterol synthesis and plays an important role in the self-feedback control of intracellular cholesterol [18, 19].

The aim of this investigation was to evaluate whether SREBP2 is regulated by estrogen and to further understand the regulatory pathway. Lipid metabolism mainly occurs in the liver, so we focused on hepatocytes to study this mechanism.

## Materials and methods

### Cell culture and ELISA assays

Human hepatoblastoma (HepG2) and hepatoma (HuH-7) cell lines were purchased from the Cell Resource Center of the Shanghai Institutes for Biological Sciences of the Chinese Academy of Sciences. These cells were cultured at 37 °C in 95% CO_2_ in high-glucose phenol red Dulbecco’s modified Eagle medium (DMEM; Gibco-BRL) supplemented with 10% fetal bovine serum (FBS; Gibco-BRL) and 100 U/ml streptomycin and penicillin (Gibco-BRL). After the cells were 40–50% confluent, the medium was replaced with phenol red-free DMEM (Gibco-BRL) supplemented with 1% charcoal-stripped FBS (Gibco-BRL). 17β-estradiol (E_2_; 7.14 nmol/l; Sigma-Aldrich), which is the most active form of estrogen [20], was added to the culture medium at gradient concentrations (0, 10^− 9^, 10^− 7^ and 10^− 5^ mol/l) for 24 h.

The concentrations of TC, TG, LDL-C and high-density lipoprotein cholesterol (HDL-C) in the culture media of HepG2 cells were measured using an ELISA kit (R&D) according to the manufacturer’s instructions.

### RT-PCR and quantitative real-time PCR analysis

Total RNA was extracted from scraped cells using Trizol reagent (Takara) and reverse transcription was performed using the protocol of the PrimeScript RT reagent Kit (Takara). PCR was carried out in a thermal cycler (PTC-200 DNA Engine; MJ Research). The RT-PCR product was visualized in a 1% agarose gel.

Real-time PCR was performed using an Applied Biosystems 7900 Fast Real-Time PCR System (Applied Biosystems). After normalizing to glyceraldehyde-3-phosphate dehydrogenase (GAPDH), the data were analyzed using the comparative threshold cycle method. Changes after treatment were noted as fold differences from untreated control values. The primer sequences for SREBP2 were 5′-GTCGGGTGTCATGGGCGGTG-3′ and 5′-CTCGCCGCTGTCGTCGATCG-3′; for 3-hydroxy-3-methylglutaryl-CoA reductase (HMGCR) were 5′-TGAGGGCTCCTTCCGCTCCG-3′ and 5′-ACTAGAGGCCACCGAACCCCG-3′; for low density lipoprotein receptor (LDLR) were 5′-TACCCCTCGAGACAGATGGT-3′ and 5′-CACTGTCCGAAGCCTGTTCT-3′; and for GAPDH were 5′-CAGGGCTGCTTTTAACTCTGG-3′ and 5′-TGGGTGGAATCATATTGGAACA-3′.

### Immunofluorescence staining

Approximately 10^4^ HepG2 cells were grown on coverslips and treated with dimethyl sulfoxide (DMSO), E_2_ (10^− 7^ mol/l), or E_2_ (10^− 7^ mol/l) + ICI (ICI 182,780, 7α-[9-(4,4,5,5,5-pentafluoro-pentylsulphinyl)nonyl]oestra-1,3,5(10)-tiene-3, 10^− 5^ mol/l; Tocris Bioscience) for 24 h. No intervention was used as a blank control.

Subsequently, the cells were fixed in 4% paraformaldehyde (Sigma) for 30 min at room temperature and then permeabilized with 0.25% Triton X-100 (Sigma) in PBS for 20 min at 25 °C. The fixed and permeabilized cells were blocked in 1% BSA and incubated overnight at 4 °C with a 1:100 dilution of anti-SREBP2 antibody (Abcam). Then the cells were incubated with a 1:200 dilution of Alexa Fluor 594-labelled mouse anti-rabbit IgG (Invitrogen) for 2 h. The nuclei were stained with 0.125 μg/ml DAPI for 15 min.

We analyzed the slides with an Olympus BX51TF fluorescence microscope (Olympus Corporation), with excitation/emission at 530/580 nm for the red fluorescence and 358/461 nm for DAPI. Finally, we analyzed the images using Image-J software from the United States National Institutes of Health (http://rsb.info.nih.gov/ij/). The intensity was calculated using the mean grey value.

### Bioinformatics analysis and chromatin immunoprecipitation (ChIP)

We used regulatory sequence analysis tools (http://rsat-new.ccb.sickkids.ca/) to analyze the sequence of the SREBP2 gene promoter to find high-score ERE-like sequences. HepG2 and HuH-7 cells were treated with 10^− 7^ mol/l E_2_ for 24 h and then cross-linked according to the Millipore EZ-ChIP Assay Kit protocol (Millipore).

Immunoprecipitation was performed with the following antibodies purchased from Millipore: mouse anti-human ERα ChIP antibody, mouse IgG used as the negative control, and mouse anti-human RNA Polymerase II antibody used as the positive control.

SREBP2 was then detected via PCR using 5′-GTCTCCAACTCCTGACCTCAA-3′ and 5′-AGTGCCTTGCATACTGCTGTA-3′ as the primer sequences. The PCR products were analyzed using agarose electrophoresis and the band was excised from the gel. Finally, the PCR product was sequenced by Invitrogen.

### Luciferase reporter assay

The putative or mutated ERE-like sequences were synthesized by GeneCopoeia and the fragments were digested with restriction endonucleases XhoI and KpnI (Thermo Fisher Scientific), which respectively recognize the sequences C^TCGAG and GGTAC^C. Then, they were ligated into the pGL3-basic plasmid (Promega). After that, we completed construction of the luciferase reporter systems of the SREBP2 promoter: SREBP2-Luciferase (SREBP2-Luc) and SREBP2-Mutation (SREBP2-Mut).

The putative ERE-like sequence was GCATTCGCTCCGAGGCCGCGGGGGGAGGGACCTCACTATGCAAATCTGAGCTGCTGATCGATGACGCGCCATCACCCCACGCACCGCTTCGCTCGCCCATTGGCTGAGATGAGCCTGGTCCCATTGACAACAAACAGGGGGGCGCGCGGCCTGGAGGCGGGGCCGCAGGGGGCGCGGGCTGGGGCGGGGGAATCCCGCCCCGCC.

The mutated ERE-like sequence was GCATTCGCTCCGAGGCCGCGGGGGGAGGGACCTCACTATGCAAATCTGAGCTGCTGATCGATGACGCGCCATCACCCCACGCACCGCTTCGCTCGCCCATTGGCTGAGATGAGCCTCCCAGCATTGACAACAAACAGGGGGGCGCGCGGCCTGGAGGCGGGGCCGCAGGGGGCGCGGGCTGGGGCGGGGGAATCCCGCCCCGCC.

HepG2 and HuH-7 cells were cultured in 6-well plates for 24 h in phenol red-free DMEM supplemented with 1% charcoal/dextran-treated FBS. Using Fugene HP transfection reagent (Roche Applied Science), the luciferase reporter plasmid containing the SREBP2 promoter and the pRL-TK reporter plasmid (cDNA encoding Renilla luciferase; Promega) were co-transfected into the cells. After 24 h of transfection, 10^− 7^ mol/l E_2_ was added for an additional 24 h. Luciferase activities in the cell lysates were measured using the dual-luciferase reporter assay system (Promega) according to the manufacturer’s instructions. After normalization to the Renilla luciferase activity, luciferase values were calculated.

### Statistical analysis

The 2-tailed Student’s t test was used to evaluate the statistical significance of the difference between two groups. One-way ANOVA and Turkey’s post hoc tests were used to evaluate the statistical significance of the difference between more than two groups. All statistical analyses were carried out using SPSS 16.0. The results were recorded as the means ± SEM and were considered significantly different at *p* < 0.05.

## Results

### Elevated SREBP2 expression, target gene expression and lipid secretion in HepG2 cells after treatment with E_2_

**Fig. 1:**
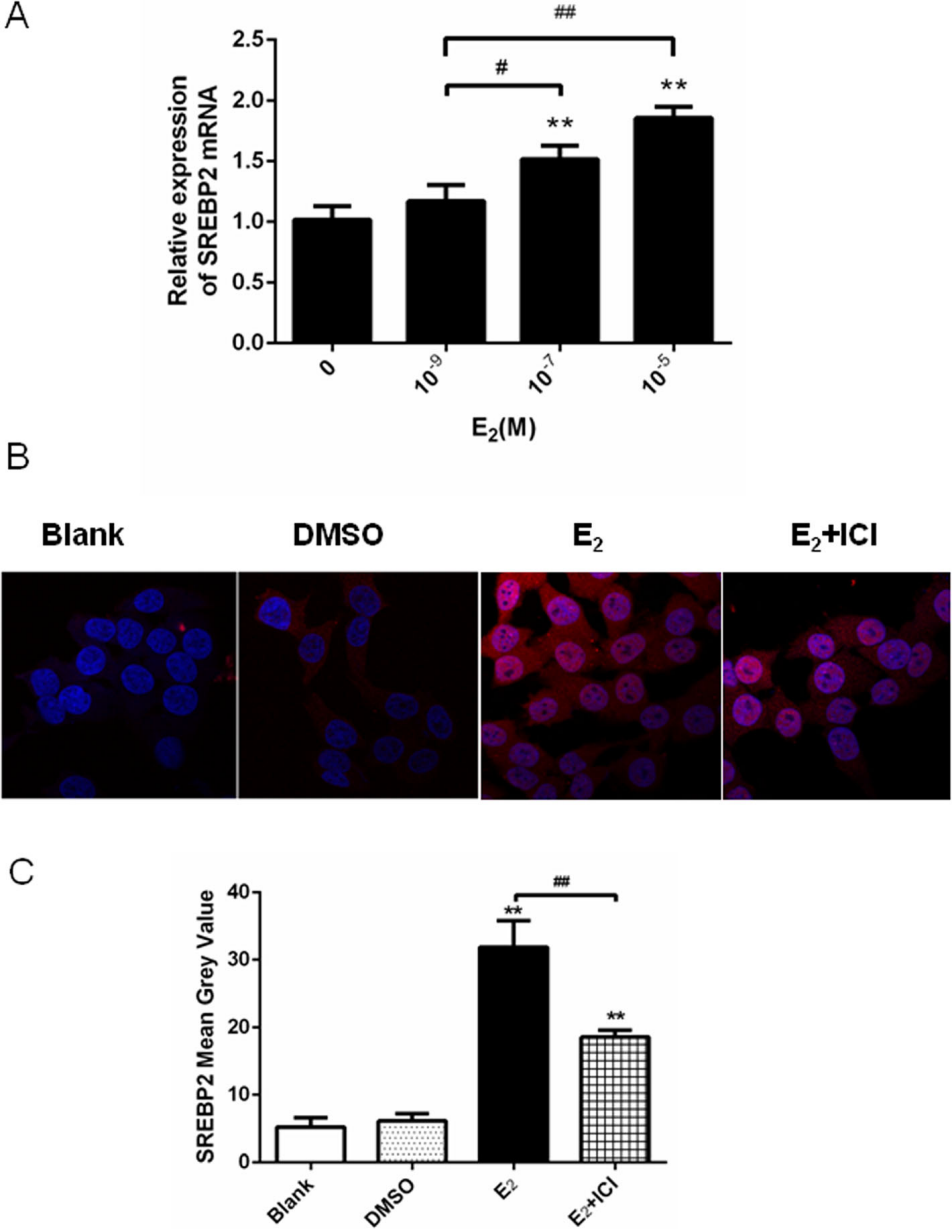
E_2_ increased SREBP2 expression in HepG2 cells in a dose-dependent manner. The effects were attenuated in the presence of ICI, an ER antagonist. **a** The SREBP2 mRNA expression in HepG2 cells after 24 h treatment with E_2_. Note the dose dependency of the change in expression. **b** The SREBP2 protein stained red in HepG2 cells after 24 h treatment with E_2_ (10^− 7^ mol/l) or co-treatment with ICI (10^− 5^ mol/l). **c** Mean gray values of SREBP2 expression. The experiments were repeated three times and data are presented as means ± SEM. *p < 0.05 and **p < 0.01 compared with the corresponding control group (a: no E_2_-treated group; c: blank group). ^#^p < 0.05 and ^##^p < 0.01 compared with the value in the E_2_-treated group. SREBP2: sterol regulatory element-binding protein; E_2_: estradiol; ER: estrogen receptor; DMSO: dimethylsulfoxide; ICI: ICI 182,780

SREBP2 mRNA expression in HepG2 cells showed a dose-dependent increase when treated with E_2_ (Fig. 1a). It was found that E_2_ at 10^− 7^ mol/l increased the expression of the SREBP2 protein in HepG2 cells compared with that in the blank control and DMSO groups (negative control; Fig. 1b). From the immunofluorescence analysis, the mean grey value of SREBP2 in the E_2_-treated group was significantly higher than that in the control group (Fig. 1c). Furthermore, the increased protein levels were obviously blocked when the cells were treated with ICI (Fig. 1b, c), which is an estrogen receptor-alpha (ERα) antagonist [21], indicating that the estrogen receptor was involved.

**Fig. 2:**
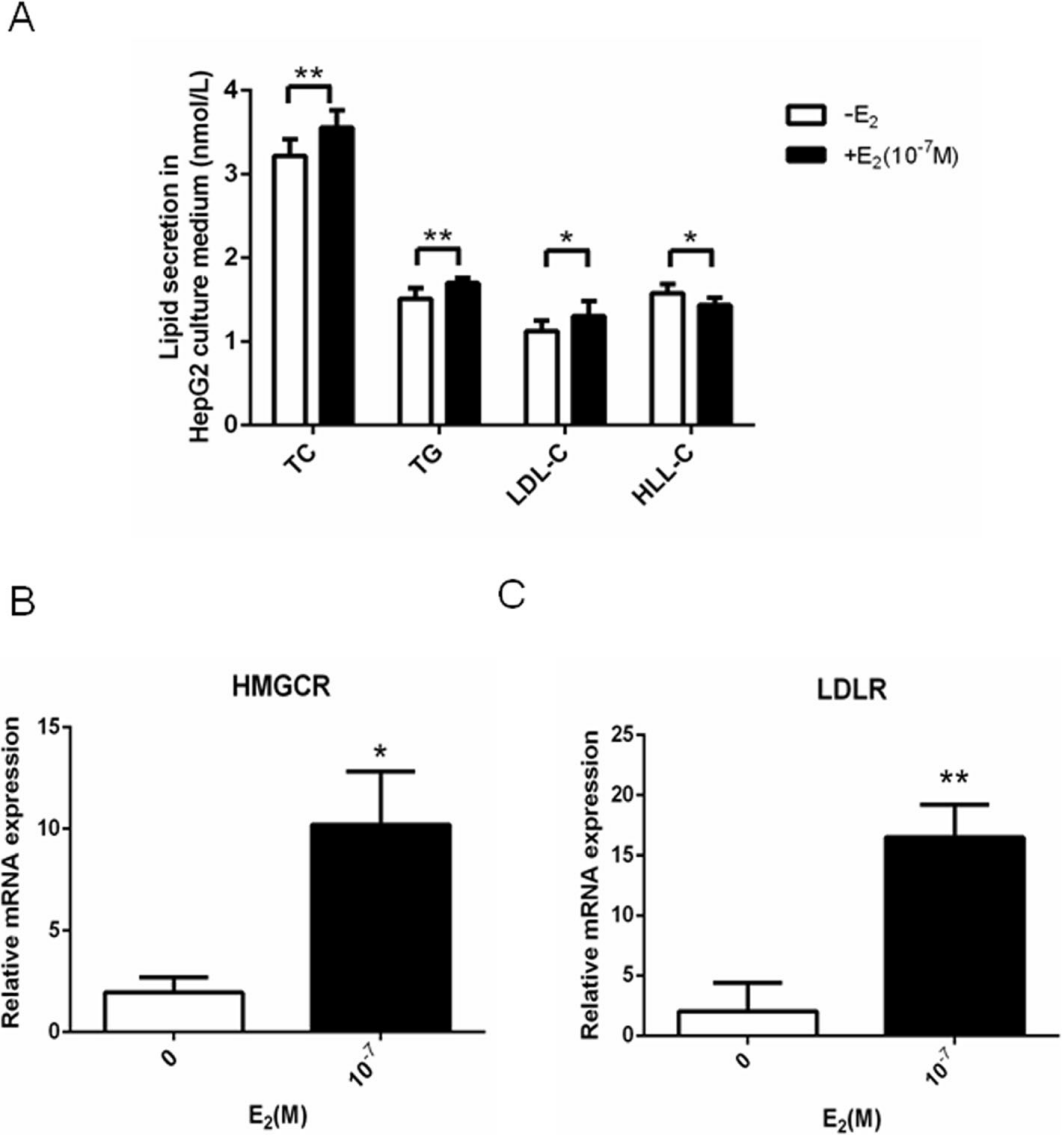
E_2_ increased lipid secretion and the expression of HMGCR and LDLR mRNA in HepG2 cells. **a** The concentration of TC, TG, LDL-C and HDL-C in HepG2 cells after 24 h treatment with E_2_ (10^− 7^ mol/l). **b** and **c** The HMGCR (**b**) and LDLR (**c**) mRNA expression in HepG2 cells after 24 h treatment with E_2_ (10^− 7^ mol/l). The experiments were repeated three times and data are presented as means ± SEM. *p < 0.05 and **p < 0.01 compared with the corresponding control group. E_2_: estradiol; TC: total cholesterol; TG: triglycerides; LDL-C: low-density lipoprotein cholesterol; HDL-C: high-density lipoprotein cholesterol; LDLR: low-density lipoprotein receptor; HMGCR: 3-hydroxy-3-methylglutaryl-CoA reductase

Furthermore, E_2_ stimulated lipid secretion in HepG2 cells, as could be seen in the increased TC, TG and LDL-C levels and decreased HDL-C levels (Fig. 2a). Increased mRNA expression of the SREBP2 downstream target gene HMGCR (Fig. 2b) and LDLR (Fig. 2c) were found in HepG2 cells after E_2_ (10^− 7^ mol/l) treatment.

### Bioinformatics analysis of the SREBP2 promoter

The promoter sequence of the SREBP2 gene was analyzed using regulatory sequence analysis tools to identify the putative EREs. The bioinformatics analysis showed that there were multiple possible ERE-binding regions in the SREBP2 promoter. The high-scoring ERE-like sequences were: GGTCCcatTGACA (− 88 ~ − 76), GATGAcatGGACA (− 1510 ~ − 1498) and GCACAcctCGGCC (− 1675 ~ − 1663).

### Identification of the predicted putative ERE in the SREBP2 promoter

**Fig. 3:**
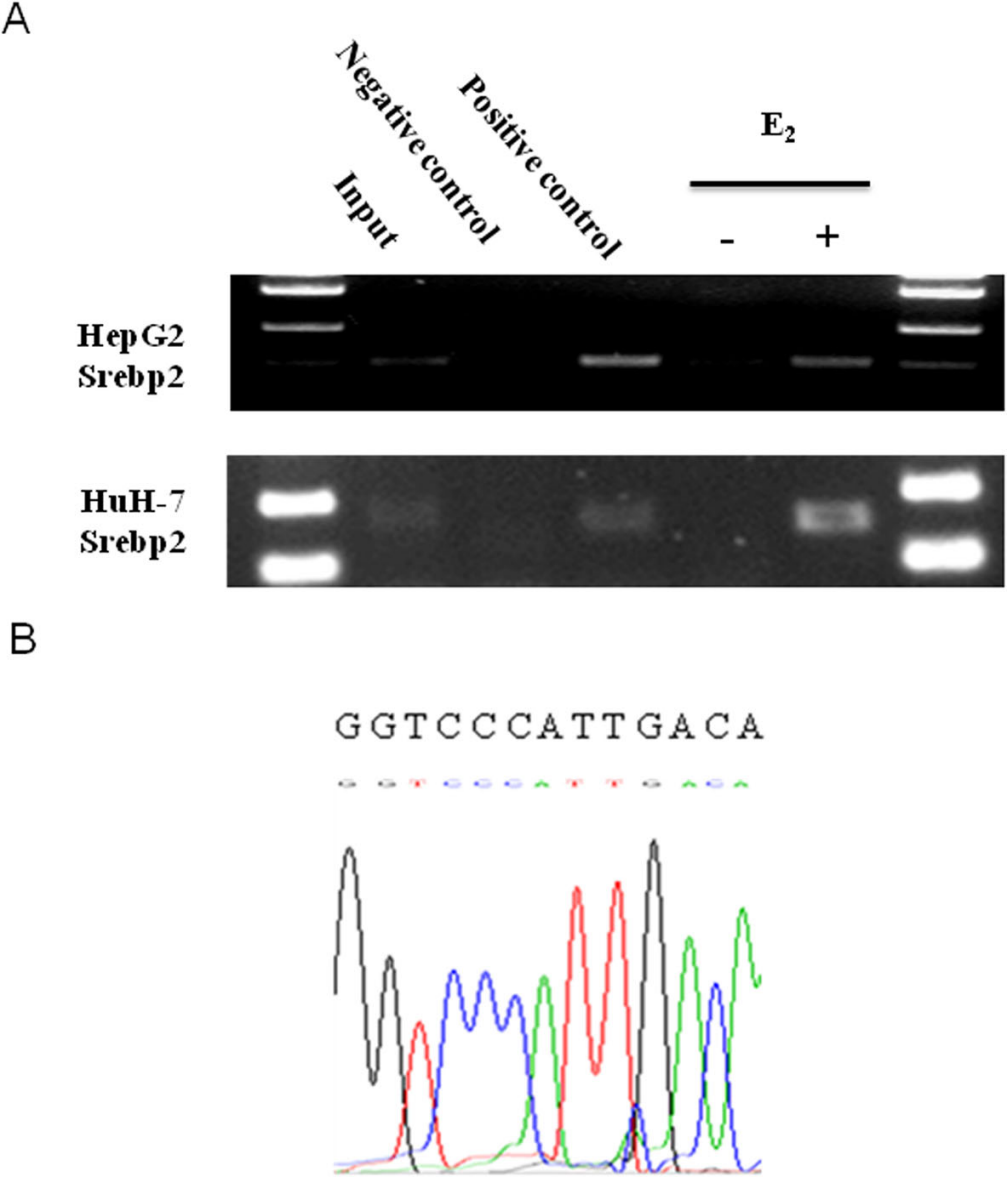
Existence of an ERE within the SREBP2 promoter. **a** ChIP analysis was performed using anti-ERα or anti-RNA polymerase II antibody to ascertain the existence of the ERE in the promoter of the SREBP2 gene. The PCR results show that one fragment containing the putative ERE could be precipitated after treatment of HepG2 and HuH-7 with E_2_ (10^− 7^ mol/l) for 24 h. **b** The pulled-down band was excised from the gel and sequenced. SREBP2: sterol regulatory element-binding protein; E_2_: estradiol; ERE: estrogen response element; ChIP: chromatin immunoprecipitation

ChIP analysis was used to confirm that SREBP2 expression is directly regulated by E_2_ through an ERE. Different primers were used to amplify the high-scoring ERE-like sequences in the promoter of SREBP2. The results show an ERE sequence at − 88 ~ − 76 of the SREBP2 promoter (Fig. 3a). Gel extraction of the PCR product was performed and sequencing revealed that the predicted ERE sequence was contained (Fig. 3b). The results suggest that one fragment containing the putative ERE (GGTCCcatTGACA) was captured using ChIP after treatment with E_2._

### Function of the putative ERE in the SREBP2 promoter

**Fig. 4:**
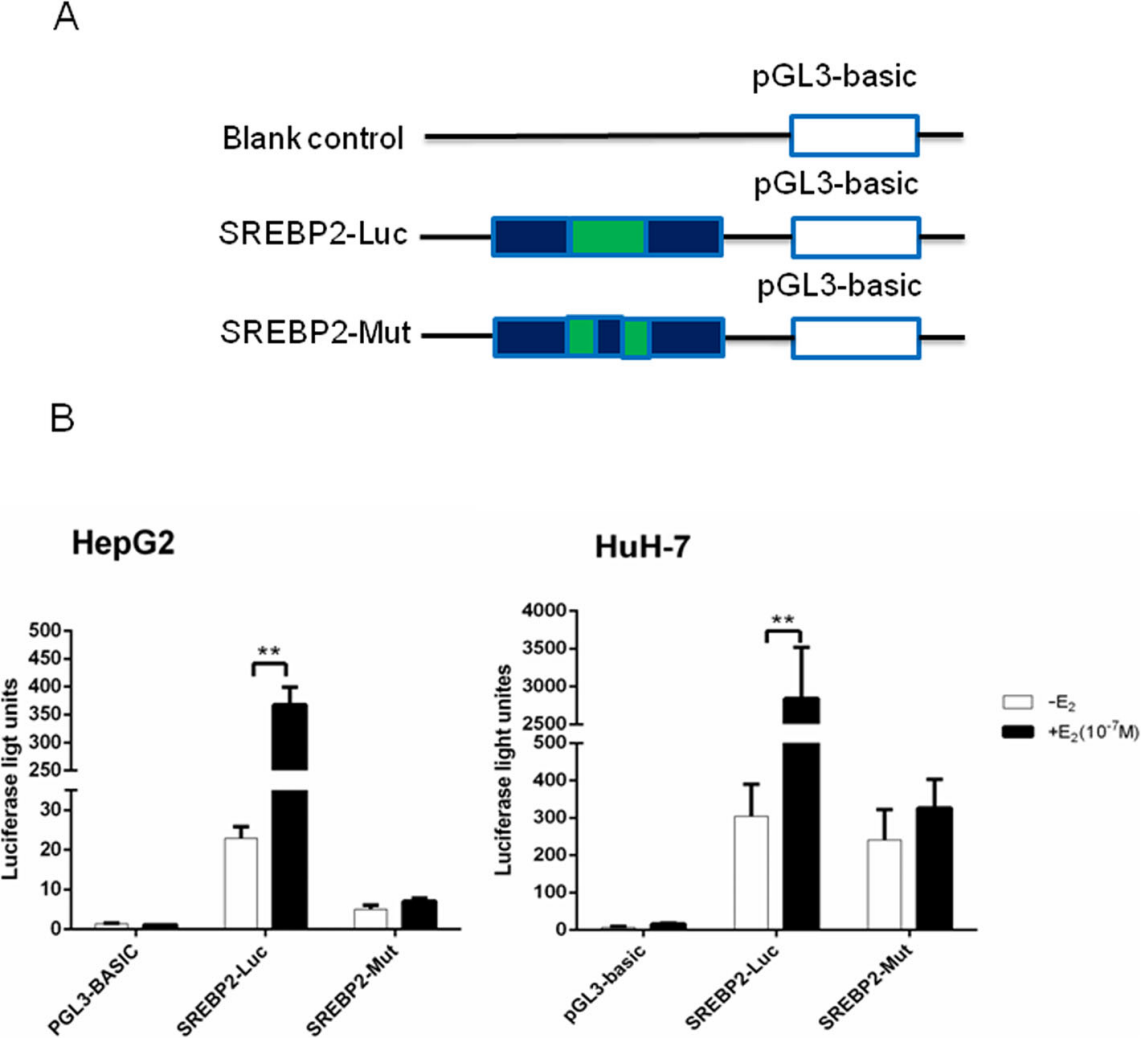
The ERE was identified functionally within the SREBP2 promoter. **a** Schematic diagram of luciferase reporter constructs. Blank control: pGL3-basic plasmid; SREBP2-Luc: pGL3-basic plasmid with the putative ERE-like sequence insert; SREBP2-Mut: pGL3-basic plasmid with the mutative ERE-like sequence insert. **b** Luciferase activities of three report systems in the presence or absence of E_2_ (10^− 7^ mol/l) were compared with each other. The experiments were repeated three times and data are presented as means ± SEM. **p < 0.01 compared with the value in the non-E_2_-treated control group. SREBP2: sterol regulatory element-binding protein 2; E_2_: estradiol

We transfected HepG2 and HuH-7 cells with luciferase reporter constructs (SREBP2-Luc, SREBP2-Mut or blank control). Luciferase activities were measured after treatment with E_2_ (Fig. 4a) to determine whether the putative ERE plays a functional role in estrogen-dependent transcriptional activation. We found that the construct of the putative ERE (GGTCCcatTGACA) could be activated by E_2_, while cells were unaffected when using the construct of the mutated element (CCCAGcatTGACA; Fig. 4b). Our results suggest that a functional ERE motif exists in the SREBP2 gene promoter, and that the ERE motif is involved in mediating estrogen-dependent SREBP2 expression.

## Discussion

In this study, we found that E_2_ could promote SREBP2 expression in HepG2 cells. Through a bioinformatics analysis of the sequence of the SREBP2 gene promoter, we predicted that there were multiple ERE-like sequences in this region. Furthermore, a functional ERE was identified in the SREBP2 promoter.

SREBPs belong to a transcription factor superfamily that can activate a series of enzymes involved in lipid biosynthesis and absorption, such as LDLR, HMGCR, HMG-CoA synthase and squalene synthase. Therefore, SREBPs play a key role in the lipid metabolic pathway [22, 23]. SREBP1a and SREBP1c are two products of the same gene, located in the 17p11.2 region [24, 25]. Produced by another gene located in the 22q13 region, SREBP2 mainly regulates the transcription of enzymes required for cholesterol metabolism [26].

It has been reported that inflammatory cytokines can aggravate cholesterol accumulation in hepatocytes by disturbing SREBP2 regulation, and that SREBP2 expression inhibition can significantly increase cholesterol accumulation [27]. In addition, SREBP2 can specifically bind to SREs on lipid-related genes, such as LDLR and HMGCR, to directly regulate and maintain intracellular cholesterol homeostasis when intracellular cholesterol is deficient [28, 29]. Thus, SREBP2 expression inhibition could disrupt SREBP2-mediated LDLR and HMGCR feedback regulation, thereby causing excess accumulation of cholesterol.

Aberrant accumulation of lipids in the arterial wall can lead to atherosclerosis, which plays an important role in the progression of cardiovascular disease [30]. Lipid disorders may easily occur in postmenopausal women due to estrogen deficiency. Low-dose estrogen supplementation can lower the cholesterol level and thus reduce the incidence and mortality of cardiovascular disease [31, 32]. However, the underlying mechanisms still need to be completely elucidated.

In this study, we found that E_2_ could regulate SREBP2 expression. This was validated by SREBP2 expression inhibition after treatment with the ERα antagonist ICI. The expression of SREBP2 mRNA significantly increased after treatment with 10^− 7^ mol/l and 10^− 5^ mol/l E_2_ compared to 10^− 9^ mol/l E_2_, which is in the physiological range for premenopausal women [33]. Furthermore, the lipid secretion content in the supernatant of the hepatic cell lines increased and the expressions of the SREBP2 downstream target genes HMGCR and LDLR mRNA were upregulated after E_2_ treatment in hepatic cell lines. Therefore, our results reveal that E_2_ may regulate lipid metabolism by affecting SREBP2 expression, and thereby relates to cardiovascular diseases.

Lipids are mainly synthesized in the liver [34], so we used hepatic cell lines to study this mechanism. HepG2 and HuH-7 cells express all three estrogen receptor subtypes: ERα, ERβ and G protein-coupled estrogen receptor (GPER) [35, 36].

Estrogen can exert multiple-pathway regulatory effects by means of acting on its receptors [37]. Classical estrogen receptors, such as ERα and ERβ, mainly exist in the nucleus and exert their functions through the classical regulatory pathway as follows. Estrogen binds with ER and induces Hsp90 to separate, thus forming ER homologous or heterologous dimers. Activated ER binds with ERE and the ER–ERE complex recruits other proteins to form a transcription initiation complex to induce transcription [12].

There has been evidence of the regulation of SREBP gene transcription as a mechanism to alter SREBP levels. There are binding sites for the thyroid hormone receptor in the 5′-flanking sequence of SREBP2 [38]. Strikingly, in this study, an ERE site was identified in the SREBP2 gene promoter. Therefore, SREBP2 regulation by estrogen provides a mechanism to link lipid disorder with estrogen.

We used a luciferase activity assay to analyze the activity of this ERE sequence and found that after E_2_ treatment, the activity of the plasmid containing this ERE sequence was significantly higher than that of the blank control or mutant sequence.

In our study, a functional ERE (GGTCCcatTGACA) was identified in the − 88 ~ − 76 region of the SREBP2 promoter for the first time. The sequence of ERE in the SREBP2 gene promoter is similar to that of typical EREs, which are elements with palindrome structure. The common sequence is 5′-GGTCAnnnTCACC-3′ (where n represents any nucleotide) and contains 13 inverted repeat base pairs and 3 bases that can be randomly replaced. However, in the genes regulated by estrogen, only a few contain the canonical ERE sequence and the majority of them have a variation of the common ERE sequence [39, 40]. Thus, our study showed that E_2_ could directly regulate SREBP2 expression via an ERE in its promoter.

**Fig. 5:**
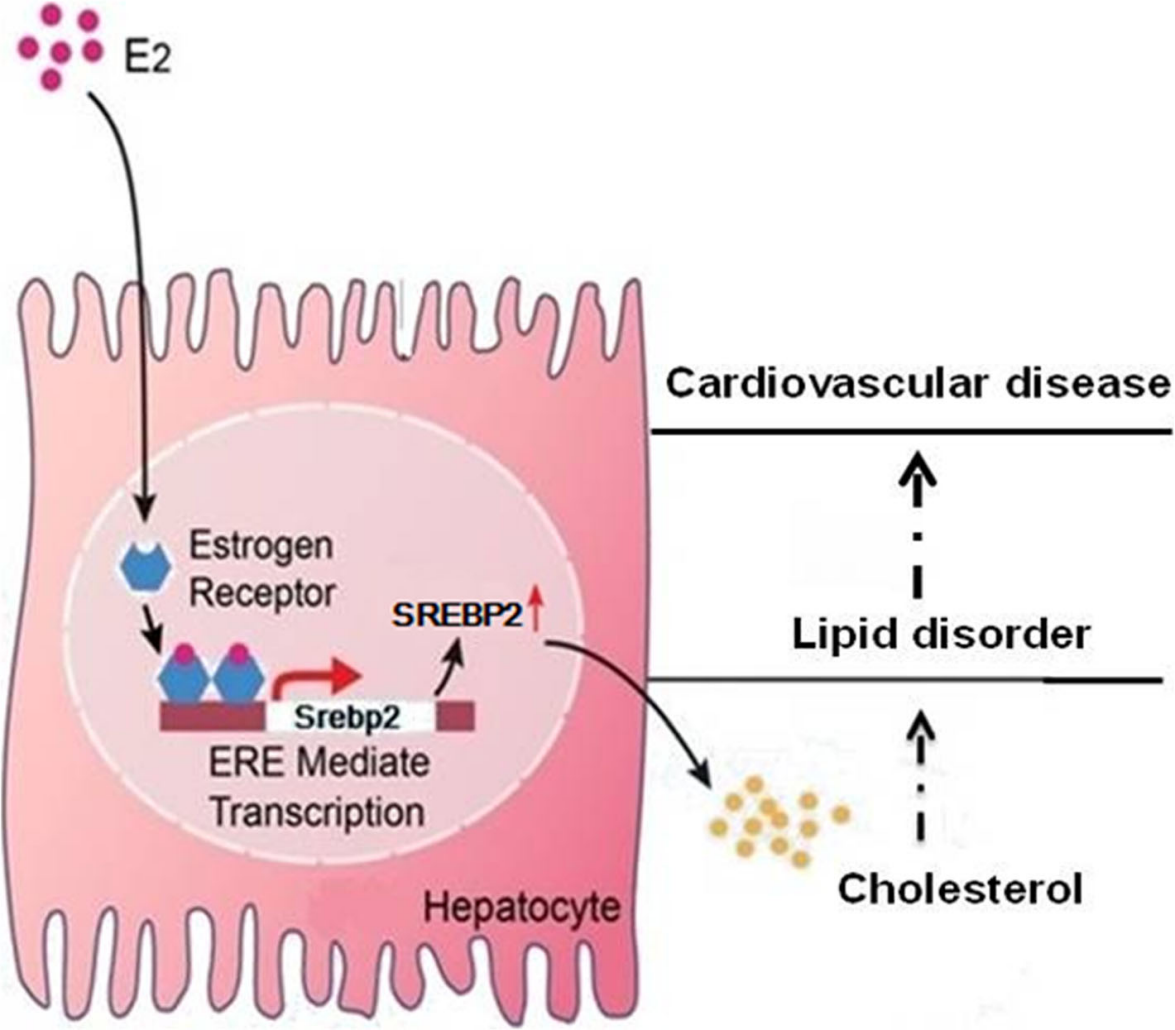
Hypothetical schematic representation. E_2_ can upregulate the SREBP2 expression in hepatocytes via an ERE in the promoter. This induces elevated levels of cholesterol that may be related to increased risk of lipid disorder and cardiovascular disease. E_2_: estradiol; SREBP2: sterol regulatory element-binding protein; ERE: estrogen response element

Our findings demonstrate that estrogen directly activates SREBP2 gene expression. An ERE, one binding site for ERα, has been identified in the 5′-flanking sequence of SREBP2. It suggests that estrogen may have an effect on lipid metabolism by regulating the SREBP2 promotor. Our results provide a basis for cardiovascular disease prevention and treatment (Fig. 5, schematic of proposed mechanism).

## Data Availability

The datasets used and/or analyzed during this study are available from the corresponding author on reasonable request (mengye@ustc.edu.cn).

## Abbreviations

ChIP: Chromatin immunoprecipitation
E_2_: Estradiol
ERE: Estrogen response element
HDL-C: High-density lipoprotein cholestero
HepG2: Human hepatoblastoma cells
HMGCR: 3-hydroxy-3-methylglutaryl-CoA reductase
HuH-7: Human hepatoma cells
LDL-C: Low-density lipoprotein cholesterol
LDLR: Low-density lipoprotein receptor
SREBP2: Sterol regulatory element-binding protein 2
TC: Total cholesterol; TG: triglycerides
